# The modular nature of protein evolution: domain rearrangement rates across eukaryotic life

**DOI:** 10.1186/s12862-020-1591-0

**Published:** 2020-02-14

**Authors:** Elias Dohmen, Steffen Klasberg, Erich Bornberg-Bauer, Sören Perrey, Carsten Kemena

**Affiliations:** 1 Institute for Evolution and Biodiversity, University of Münster, Hüfferstrasse 1, Münster, 48149 Germany; 2 Institute for Bioinformatics and Chemoinformatics, Westphalian University of Applied Sciences, August-Schmidt-Ring 10, Recklinghausen, 45665 Germany

**Keywords:** Protein domain, Rearrangement rates, Proteome analysis, Evolutionary history, Ancestral reconstruction

## Abstract

**Background:**

Modularity is important for evolutionary innovation. The recombination of existing units to form larger complexes with new functionalities spares the need to create novel elements from scratch. In proteins, this principle can be observed at the level of protein domains, functional subunits which are regularly rearranged to acquire new functions.

**Results:**

In this study we analyse the mechanisms leading to new domain arrangements in five major eukaryotic clades (vertebrates, insects, fungi, monocots and eudicots) at unprecedented depth and breadth. This allows, for the first time, to directly compare rates of rearrangements between different clades and identify both lineage specific and general patterns of evolution in the context of domain rearrangements. We analyse arrangement changes along phylogenetic trees by reconstructing ancestral domain content in combination with feasible single step events, such as fusion or fission. Using this approach we explain up to 70% of all rearrangements by tracing them back to their precursors. We find that rates in general and the ratio between these rates for a given clade in particular, are highly consistent across all clades. In agreement with previous studies, fusions are the most frequent event leading to new domain arrangements. A lineage specific pattern in fungi reveals exceptionally high loss rates compared to other clades, supporting recent studies highlighting the importance of loss for evolutionary innovation. Furthermore, our methodology allows us to link domain emergences at specific nodes in the phylogenetic tree to important functional developments, such as the origin of hair in mammals.

**Conclusions:**

Our results demonstrate that domain rearrangements are based on a canonical set of mutational events with rates which lie within a relatively narrow and consistent range. In addition, gained knowledge about these rates provides a basis for advanced domain-based methodologies for phylogenetics and homology analysis which complement current sequence-based methods.

## Background

Functional adaptations of proteins have often been observed to be caused by point mutations changing amino acids at crucial positions. These mutations typically result in altered specificity or stability of a protein. Although this process is important for evolutionary adaptations, point mutations often result in only minor changes of a protein. For greater functional changes or innovation, more drastic modifications are necessary that do not rely on numerous mutations.

Molecular mechanisms like crossing over, alternative splicing and transposition through mobile elements can cause mutational events that rearrange larger DNA fragments and therefore also alter larger regions at the protein level. Examples of such mutational events, which rearrange gene content, are for example fusion and fission. All these events lead to rearrangements that can be easily tracked at the level of protein domains, since domains are well characterised in many databases (e.g. in the *Pfam* [1] or *Superfamily* [2] database) and represent reusable structural and functional units.

The total number of defined domains is relatively small and is growing only slowly. For example, the *Pfam* domain database [1] defines about 18,000 domains in its current version (version 32). On the other hand, the number of known unique domain arrangements - defined by the linear order of domains in an amino acid sequence [3] - is much larger and growing rapidly [4]. Accordingly, rearrangements of existing domains can help explain the vast protein diversity we observe in nature [4–9].

Several studies have shown that domain rearrangements are essential in the evolution of pathways, signalling networks and cellular components. The evolution of the extracellular matrix in metazoans [10] as well as the blood coagulation cascade [11] are examples in which the reuse of domains in different contexts are considered crucial steps. Additionally, domains have been identified to play an important role in signalling networks [12] or their recombination to new arrangements in T-Cell development [13]. Lees et al. [14] showed the importance of domain arrangement changes in cancer genome evolution. Therefore, it is crucial to analyse domain changes when studying both genome evolution and specific protein families.

First attempts to study general evolutionary domain patterns focused mainly on emergence and loss of single domains [15, 16] or domain repeats [17, 18]. Later, quantitative analyses in plants and insects [19, 20] over time-scales of several hundred million years revealed hot-spots of rearrangement events at specific nodes in the phylogenetic tree. Both these studies took into account four different types of rearrangement events: fusion, fission, terminal addition and terminal loss. Together, these events are sufficient to explain a large proportion (60%-70%) of the new domain arrangements considered in those studies.

Based on these four single step events, rearrangement rates for a set of 29 plant species (dating back as far as 800 my [19]) and 20 Pancrustacean species (dating back 430 my [20]) were determined in previous studies.

In this study we use expanded species sets (up to 72 species per phylogenetic clade) to detect common patterns of domain evolution and consider several thousand more arrangements per clade compared to the two previously mentioned studies. In total, domain arrangements in five different eukaryotic clades (vertebrates, insects, fungi, monocots and eudicots) are analysed. For the first time, the results can be directly compared between these clades, since exactly the same methodology was applied to all of them.

Previously, methods were applied that had used either overlapping definitions for rearrangement events, or that analysed domain loss and emergence (e.g. [16]) separately from rearrangement events (e.g. [20]). In this study, we combine these methodologies in one consistent model, allowing us to distinguish six different single step events, thereby analysing the molecular mechanisms leading to protein innovation at unprecedented accuracy. The incorporation of additional clades and a higher number of species ensures the integrity of the observed events, for example by minimising annotation biases. The resulting rearrangement frequencies are directly comparable across the different eukaryotic clades and thus reveal the fundamental mechanisms of functional rearrangements in eukaryotes, in addition to lineage specific trends.

Furthermore, we infer functional implications of the new arrangements via *Gene Ontology* (GO) [21] term enrichment. Finally, we discuss how our methodology can be used to complement existing methods for example in phylogenetic reconstruction, by incorporating data on domain rearrangements.

## Results

To be able to draw reliable conclusions about universally valid mechanisms in protein evolution, it is necessary to ensure that a sufficient number of observable rearrangements can be explained by the six different rearrangement events defined in this manuscript (*fusion*, *fission*, *terminal loss/emergence* and *single domain loss/emergence*; see Methods). For this purpose we reconstructed the ancestral domain content and arrangements at all inner nodes of the phylogenetic trees of five eukaryotic clades (vertebrates, insects, fungi, monocots and eudicots). For all domain arrangements that differ from the parental node, we examined whether the change could be explained uniquely by one of the six events.

**Fig. 1:**
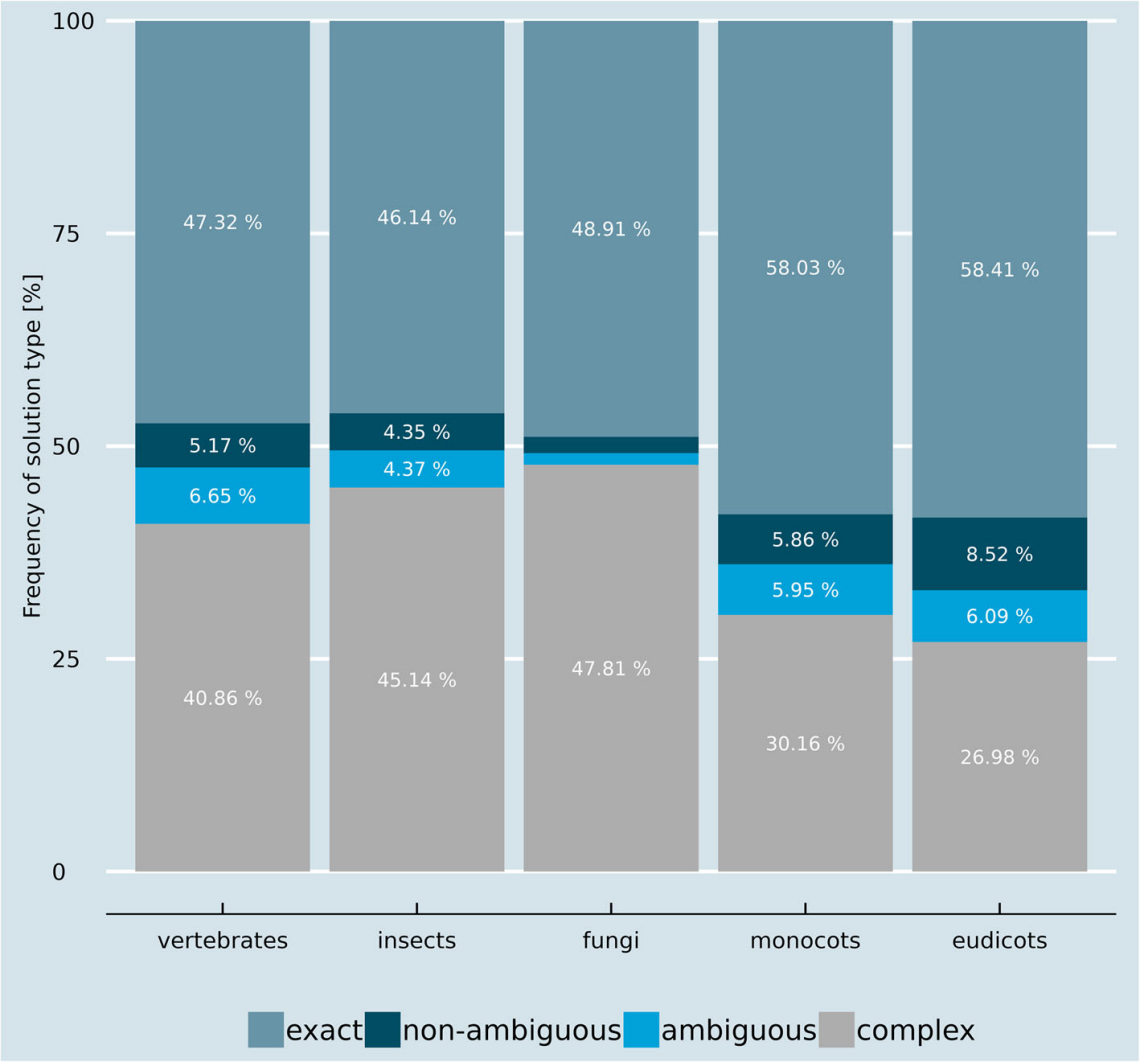
Frequency of the different solution types. Exact and non-ambiguous solutions can be found in about 50% of the cases

*Unique solutions* are either *exact solutions*, where only a single event can explain the arrangement change, or *non-ambiguous solutions*, where multiple events of the same type can explain a new arrangement (e.g. ABC: A+BC / AB+C). Only unique solutions were further analysed in detail to focus on changes which can be explained with certainty (Additional file 2). Unique solutions can explain 50% to 70% of all observed new arrangements, depending on the analysed phylogenetic clade (Fig. 1).

However, there is a small percentage of new arrangements which can be explained by multiple different event types, i.e. *ambiguous solutions* (e.g. ABC: ABC-D / AB+C). Beside these ambiguous solutions, some new arrangements cannot be explained by the defined single step events. These so-called *complex solutions* (25%-50%), would require several successive single step events.

### Comparison between clades

One major goal of this study is to find, beside clade-specific differences, universally valid evolutionary mechanisms of protein innovation that are present in all clades. Therefore, we analyse whether common patterns in domain rearrangements can be observed by measuring the relative contributions of each rearrangement event and compare them between the different clades (see Table 1 and Additional file 4).

**Table 1:** Frequencies of the six rearrangement events (in %)

	Vertebrates	Insects	Fungi	Monocots	Eudicots
Fusion	32.45	41.52	29.35	64.43	58.22
Fission	19.57	17.21	8.80	12.21	16.28
Terminal loss	20.52	19.21	16.46	10.59	13.00
Terminal emergence	0.13	0.36	0.76	1.01	0.48
Single loss	26.71	19.99	40.74	9.83	10.20
Single emergence	0.61	1.71	3.89	1.93	1.82

The percentage of fusion events in our study ranges from 29% in fungi to 64% of all observed events in monocots. Only in fungi, fusions represent not the most frequent event type, but single domain loss is most frequent. Furthermore, in all clades except fungi, fissions and terminal losses account for a similar percentage of all domain rearrangements. In fungi, loss of terminal domains accounts for twice as many rearrangements as fissions. The exceptional distribution of event frequencies in fungi compared to the other clades is discussed below.

The very low contributions of the two emergence categories, terminal and single domain emergence, of only 0.13% to 3.89% show that domain emergence is indeed rare compared to a much higher number of domain rearrangements and losses.

We observed three general patterns of the ranks of rearrangement events corresponding to the taxonomic kingdoms of animals, fungi, and plants. In the first pattern, observed in animals (i.e. vertebrates and insects), the most frequent domain rearrangement event is domain fusion (32% and 42% of rearrangements respectively), followed by single domain loss (27% and 20%) and terminal domain loss (21% and 19%). Arrangement gain by fission is slightly less common (20% and 17%), but still more frequent than the very low rates of single domain emergence (0.6% and 1.7%) and terminal emergence (0.1% and 0.4%).

The functional analysis of gained arrangements in insects (Additional file 5) using GO term enrichment reveals olfaction related adaptations (represented by GO terms of ’sensory perception of smell’, ’olfactory receptor activity’ and ’odorant binding’) are overrepresented in insects. Other overrepresented GO terms include ’sensory perception of taste’ and ’structural constituent of cuticle’.

We did not find expansions of vertebrate specific GO terms at the root of vertebrates. However, we found overrepresented GO terms related to binding (e.g. ’protein binding’, ’nucleic acid binding’) and terms related to signal transduction (Additional file 6).

The distribution and rank of rearrangement rates in Fungi (Additional file 7) resemble those of animals, with the only qualitative difference being that single domain losses were more frequent than fusions. A more detailed analysis of this phenomenon can be found below.

The third pattern of arrangement changes is observed in plants, i.e. monocots and eudicots. As in metazoans, but with an even higher percentage, the majority of new arrangements is explained by fusion (64% and 58%). The fission of one arrangement into two new arrangements is the second most frequent mechanism (12% and 16%) followed by slightly smaller numbers of terminal (11% and 13%) and single domain loss (10% and 10%).

**Fig. 2:**
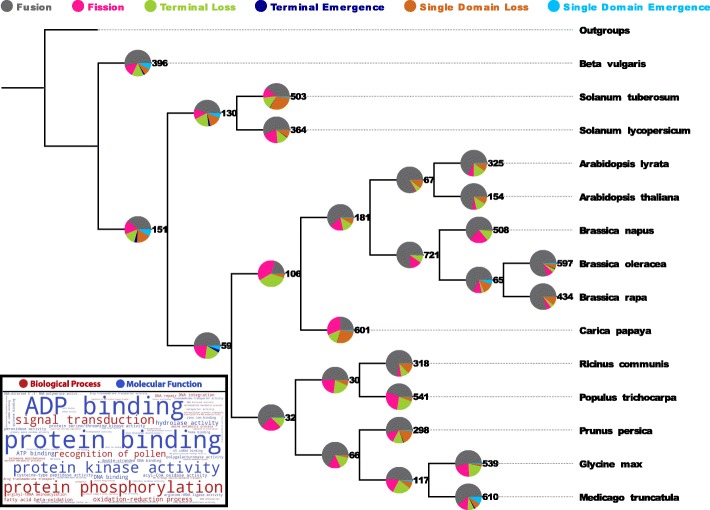
Number of rearrangement events across the eudicot phylogeny. Digit representation of the total number of rearrangement events at a specific node is indicated next to the pie chart. For details on ’Outgroups’ see Methods. Significant GO terms in gained domain arrangements are shown in a tag cloud (box). GO terms that might point to eudicot specific evolution are: ’recognition of pollen’ and ’plant-type cell wall organization’

Some GO terms are enriched in gained arrangements at the root of both plant clades that might be related to plant development and evolution, i.e. ’recognition of pollen’ in both plant clades or ’plant-type cell wall organization’ in eudicots (Fig. 2 and Additional file 8).

### Domain loss in fungi

We analysed the distribution of domain arrangement sizes in the five clades (see Additional file 9) to find possible explanations for the different patterns of event frequencies mentioned above. The results show that a strikingly high number of fungal domain arrangements consists of just a single domain and their arrangements are generally much shorter compared to vertebrates or insects. Both plant clades, monocots and eudicots, also have much shorter domain arrangements than the metazoan clades.

We found that both plant clades show the highest copy number of domain arrangements. Eudicots have 5.79 copies on average per single domain arrangement per species, while monocots have 5.64. This high number of duplications of the same domain arrangement could be explained by multiple whole genome duplications in these clades. Vertebrates follow with 1.93 copies per single domain arrangement and finally insects (1.27), while fungi show the lowest duplication count (1.15).

### Effects of domain rearrangements

The general rates of rearrangement events and their distribution in a given phylogenetic tree can provide an insight into the evolutionary history of a whole clade as well as general adaptational processes in certain lineages. However, by taking a more detailed look at the specific domains involved in the rearrangement events at specific time points, we can trace back some major steps in the evolutionary history of the studied species. Here, we show three examples of new or outstanding functions at specific nodes in the evolution of vertebrates, plants and insects which can be related to the emergence of new domains or domain arrangements.

#### The origin of hair and adaptations of the immune system in mammals

One remarkable pattern in the distribution of rearrangement events in the vertebrate phylogeny is the high rate (33%) of single domain emergences at the root of all mammals. This represents the highest percentage of single domain emergences at any node in the vertebrate tree. A closer investigation of the function of these emerged domains shows that ∼30% of the emerged domains (domains of unknown function excluded) are associated with hair. This finding is a strong signal for the origin of hair or fur, respectively, in the common ancestor of all mammals.

One of the most important structural protein families of mammalian hair is the keratin-associated protein family (KRTAPs). Hair keratins are embedded in an inter-filamentous matrix consisting of KRTAPs located in the hair cortex. Two major types of KRTAPs can be distinguished: high-sulfur/ultra-high-sulfur and high-glycine/tyrosine KRTAPs [22]. Three of these high-sulfur proteins can be found in the set of emerged domains as ’Keratin, high sulfur B2 protein’ (Pfam-ID: PF01500), ’Keratin-associated matrix’ (PF11759) and ’Keratin, high-sulphur matrix protein’ (PF04579). The proteins are synthesised during the hair matrix cell differentiation and form hair fibres in association with hair keratin intermediate filaments. Another domain that can be found in this set is the ’PMG protein’ (PF05287) domain, which occurs in two genes in mice (PMG1 and PMG2) that are known to be expressed in growing hair follicles and are members of a KRTAP gene family [23]. PMG1 and PMG2 are additionally involved in epithelial cell differentiation, while a further member of the emerged domains - ’KRTDAP’ (PF15200) - is a keratinocyte differentiation-associated protein. Keratinocytes are a cell type of the epidermis, the layer of the skin closest to the surface [24]. The KRTDAP related gene was isolated in rats between skin of prehair-germ stage embryos and hair-germ stage embryos, and shows high expression in regions of the hair follicle [25]. We can infer that the emergence of hair and fur also involved adaptation and restructuring of the skin, resulting in novel skin cell types and cell differentiation regulation mechanisms. Furthermore, the skin, and keratinocytes in particular, act as a first barrier against environmental damage and pathogen infestation and are therefore related to the second barrier, the immune system. Indeed, immune system related domains are the second biggest group in these emerged domains (>20% of domains with known function). As an example, the ’Interleukin’ domain (PF03487) emerged at the root of mammals and is associated with a group of secreted proteins and signalling molecules. The mammalian immune system is highly dependent on interleukins with certain deficiencies linked to autoimmune diseases and other immune system defects [26]. ’Lymphocyte activation family X’ is a domain also found in this set (PF15681), which is membrane-associated and expressed in B- and T-cells in addition to other lymphoid-specific cell types [27]. Additionally, out of all events occurring at the root of mammals, ’regulation of lymphocyte activation’ is an overrepresented term in the GO term enrichment analysis (see Additional file 10). These results reinforce the importance of the immune system for the early evolution of mammals.

#### Resistance to fungi in wheat

The functional analysis of gained domain arrangements using GO terms revealed an interesting pattern for the node leading to *Triticeae* which includes the two wheat species *Triticum urartu* and *Triticum aestivum* as well as the grass species *Aegilops tauschii*. Five out of the 15 enriched GO terms in *Triticeae* can be related to resistance to fungal pathogens via three different mechanisms. Chitinases are enzymes, which are known to be involved in plants’ fungal resistance and have been extensively studied in wheat species [28, 29]. The ability of these enzymes to degrade chitin, a primary component of fungal cell walls, can lead to the lysis of fungal cells and therefore provide resistance against them. We found the three significant GO terms ’chitin catabolic process’, ’cell wall macromolecular catabolic process’ and ’protein phosphorylation’ related to chitinases, which explain the innate fungal resistance of wheat and can also be utilized in genetic engineering to enhance fungal resistance in other crop plants [30]. The GO term ’protein kinase activity’ and the underlying Serine Threonine kinase has also been shown to be used in plants’ defense to fungi [31]. Another mechanism of fungal resistance is based on an ATP-binding cassette transporter, which is used in many crop plants [32]. We relate the GO term of ’ATP binding’ to this function of fungal resistance. Overall, the gained arrangements in *Triticeae* can be linked to the increased resistance of this clade to fungal pathogens.

#### Eusociality in bees

We found an example of interesting GO terms enriched at a node in *Apidae*, i.e. in the last common ancestor of the honey bee *Apis mellifera* and the bumblebee *Bombus terrestris*. This node marks one of the transitions of solitary bees to eusocial bees [33]. The overrepresented GO terms that relate to the evolution of eusociality comprise ’embryonic morphogenesis’, ’insulin-like growth factor binding’ and ’regulation of cell growth’ [33] and are additionally expanded in the species *Bombus terrestris* and *Apis cerana*. Insulin and insulin-like signalling (IIS) pathways have been shown to be differently expressed between castes in the honeybee and play a role in caste differentiation [34, 35]. Additionally, IIS modifies the behaviour of honey bee workers in foraging [36]. Functions of some domains that are associated with overrepresented GO terms can possibly be related to the emergence of eusociality, either by being involved in development or have been shown to be differentially expressed in different castes. Two domains are associated with growth factors, ’Insulin-like growth factor binding’ (PF00219) [34, 35] and ’EGF-like domain’ (PF00008). Epidermal growth factor (EGF) has been shown to be involved in caste differentiation in the honey bee by knockdown experiments [37, 38]. Several domains have been found to be differentially expressed in queens and workers in the honey bee and might be related to eusociality [39], i.e. ’Fibronectin type III domain’ (PF00041), ’Protein kinase domain’ (PF00069), ’Myb-like DNA-binding domain’ (PF00249) and ’Insect cuticle protein’ (PF00379). ’Insect cuticle protein’ is also suspected to play a role in the transition from solitary to eusocial bees [40].

## Discussion

In comparison to previous studies we can verify some of the key findings like fusions being the most common event type accounting for new domain arrangements [19, 20, 41]. At the same time we can show to what extent these findings also apply to other phylogenetic clades or where differences exist (e.g. single domain loss being the most common event type in fungi). Comparing the data basis of this study to previous ones reveals that the total number of events with a unique solution (Additional file 3) is much higher than in any previous study, while the proportion of considered solutions in other studies is similar to ours. The underlying total numbers in previous studies sum up to only a few thousand unique solutions (∼5200 in Moore’s pancrustacean set [20]) compared to ten thousands in this study (∼24250 in the insect set, which also contains 18 out of 20 of Moore’s pancrustacean species).

This increasing total number of resolvable events, while representing constant proportions over time, suggests that with increasing quality of sequences, annotations and motifs in databases we are able to explain more of the evolutionary history, but at the same time add more unknown or complex cases. However, the ambiguous and complex solutions we find in this study can be resolved to some extent with further investigation and approaches specific for this problem. In some cases, the ambiguity of ambiguous solutions might be resolved by computing domain trees based on the primary sequences. This is, though, outside the scope of this study and the information gain would be minimal as only a very low percentage (∼5%) of all solutions are ambiguous ones.

Complex solutions might be resolved with the use of a deeper and denser phylogeny. Such a phylogeny might provide additional inner nodes which are required to be able to track the arrangement changes using single steps. Another potential way to resolve the underlying molecular rearrangement events of complex gains could be to consider not only single step events, but also solutions with two or more steps. However, the latter approach would strongly increase the complexity of the calculations, while at the same time introducing uncertainty by introducing multiple additional ambiguous solution possibilities.

The GO term enrichment analysis based on domain changes during evolution can give additionally useful insights into major functional adaptations of a clade. In insects for example all described enriched GO terms (’sensory perception of smell’, ’olfactory receptor activity’, ’odorant binding’, ’sensory perception of taste’ and ’structural constituent of cuticle’) are essential for communication between individuals, for example to find mating partners by sensing pheromones over long distances or to tell nest mates from potential enemies in social insects [42–44]. For the fungi clade enriched terms are ’carbohydrate metabolic process’ and ’cellulose binding’, which can be seen as important adaptations for the lifestyle of some fungal species. Many fungal species (e.g. Serpula lacrymans) are wood-decaying, for which both metabolic functions are crucial. Another hint for the wood-decay related background of these adaptations could be the enriched GO term ’oxidation-reduction process’, which can be associated to lignin deconstruction as well as to cellulose/xylan degradation.

One evolutionary mechanism of specific interest is loss of function as a process of adaptation. In this study especially the different signals for losses in plants and fungi are worth a more detailed investigation. In plants the high rates of fusion and fission and low rates of losses can be related to plant specific genome properties. Transposable elements play a major role in plants by the frequent creation of retrocopies and thus contribute to a high number of observable gene duplications in plants [45–47]. Additionally, many whole genome duplications have been observed in plants, leading to large genomes as a basis for rearrangements while maintaining the original gene and function [47–49].

A possible explanation for the high frequency of single domain loss in fungi could be the generally high fraction of single domain arrangements in their proteomes. Such a high fraction of single domain loss is however not observed in plants, although eudicots also have a high fraction of short domain arrangements, comparable to that of fungi (Additional file 9). The difference between eudicots and fungi regarding single domain losses can be explained via the average copy number of single domain arrangements in both clades. The results of the duplication count analysis imply that fungi possess by average just one copy (1.15) of every single domain arrangement, which can explain the high amount of single domain losses observed in this clade, while eudicots possess by average 5-6 copies (5.79). From a functional perspective there is evidence that gene loss plays a particularly important role in fungi. In fungi, massive gene loss as a major evolutionary mechanism has been linked to biotrophy to discard dispensable genomic components [50] and to adaptations to new hosts [51]. In addition to some biotrophic species in our fungi dataset, such as *Puccinia graminis* [52] or *Ustilago maydis* [53], there are other species for which host adaptations or biotrophy cannot be the explanation for large-scale gene loss, since they are not biotrophic, like *Saccharomyces cerevisiae*. However, for *Saccharomyces* species there is evidence for an ancient whole genome duplication event followed by massive gene loss (an estimated 85%) of the duplicated genes [54]. Next to whole genome duplication, other studies also linked polyploidy in fungi and plants to high loss rates [55]. In contrast to plants, where whole genome duplication events appear to lead to a high copy number of domains, fungi seem to possess mechanisms to rapidly reduce their genome size and throw out redundant or unnecessary information. The examples suggest that the unusually high rate of single domain losses observed in the fungi clade are the result of a fungi-specific evolutionary mechanism of genome evolution involving gene loss as a major driving force. In conclusion, next to genomic properties such as the abundance of duplicates as a basis for subsequent changes other factors likely play important roles for the evolutionary distribution of certain rearrangement events. These factors can be as described differences in lifestyles, but also differences in reproduction patterns are potential candidates, as the presence/absence of sexual reproduction in many plant and fungal species can provide an explanation for the observed differences in these clades.

## Conclusions

### Robustness of results and methodological limitations

Overall, this study shows that only six different basic event types are sufficient to explain the majority of new domain arrangements contributing to the complex process of protein innovation in major phylogenetic clades. The results are highly consistent across all major clades, i.e. similar proportions of arrangements can be explained by the same events across all clades, suggesting that misannotations do not bias the outcome significantly and the findings can be considered to be universally valid across eukaryotes. Furthermore, the similar distribution of events in insects and eudicots, representing 50% and 70% uniquely resolved events in the corresponding clade, suggests that unresolved events in all clades are likely a matter of resolution of the tree and not changing the distribution of events observed in this study. Additionally, the results of the conducted jackknife test (see Additional file 4) make sampling biases unlikely.

However, this study focuses on phenotypic changes through mutational events, which are observable solely on a domain level. Many of the investigated event types can be caused by different molecular mechanisms on the DNA level, which rates can vary compared to each other and be influenced by lifestyles or reproduction patterns. For a more complete picture of the evolutionary history, domain-based methods such as the here presented one, should be therefore complemented with primary sequence-based methods to answer specific biological questions.

### Future implications and perspectives

Domain-based approaches have some special properties compared to primary sequence-based ones, making them particularly suited for different types of analyses. A general difference of domain-based approaches is the use of a larger alphabet with fewer letters per sequence. Additionally, changes on the domain level are less frequent than mutations of amino acids or nucleotides, why domains are especially suited for long time scales. The high conservation of domains and a high sensitivity in detection via their underlying Hidden Markov Models enable the accurate detection of homologous sequence fragments even in highly diverged sequences. Therefore, domain-based approaches avoid problems of primary sequence-based methods as in homology detection. Also, for phylogenetic analyses there are certain advantages such as reduced biases through saturation or long branch attraction.

Still, multiple parameters and properties for domain rearrangements are unknown, limiting the possibilities for practical implementations of domain-based approaches. Unfortunately, no general rates and transition probabilities for domain rearrangement events were known before this study that could be applied to diverse and bigger data sets. Also time depths for all phylogenies and branches are not resolvable by now. Despite these limitations, the parsimony approach used in this study can map the changes across different speciation events in the tree and shows no significant bias introduced by the method. In fact, as demonstrated in this study, domain rearrangement rates hardly depend on depths of single nodes in the phylogenetic tree, suggesting the here used parsimony approach seems to be accurate and resulting in feasible and substantiated basic rearrangement rates. In a next step these estimated rates can lay the foundation for more advanced domain-based methods, while this further step cannot be provided by this study on its own already. It should be noticed that the here estimated rates and frequency of events are the raw descriptive numbers to provide an unbiased data basis, but for advanced methods these should be carefully normalised dependent on the scope of application. The available number of proteins in a proteome as well as the frequency of duplication events and therefore active mobile elements in a genome are for example influencing factors for domain rearrangements and should be taken into account. Additionally, emergence and loss events in this study are seen from a functional perspective and the presence or absence of an arrangement in the protein repertoire is of main interest, while we do not consider expansions or contractions of the same arrangement through copy number.

Summarising, this study is meant to elucidate the dynamics of domain rearrangements in different taxonomic groups and by doing so providing a data basis for more advanced methods. Analyses from a domain point of view could complement other methods and make it easier to estimate biases of other studies or overcome certain limitations. In conclusion, the results of this study demonstrate the high potential of domain-based approaches, while at the same time providing a basis for further development in this field.

## Methods

### Data set preparation

Five data sets are analysed in this study, each representing a different phylogenetic clade: vertebrates (61 species), insects (72), fungi (36) monocots (19) and eudicots (14) (see Additional file 11). Only proteomes are included that have a DOGMA [56] quality score ≥ 75%, to ensure that all proteomes used are of high and similar quality. This prevents the calculation of unduly high number of rearrangement events due to poor genome and gene prediction quality. To assure better comparability between the clades and the species within a clade, the corresponding ensembl database [57] as a widely used source for comparative genomics, was screened primarily for proteomes when available (fungi, plants(eudicots and monocots) and vertebrates).

As outgroups, a set of five well-annotated species (*Arabidopsis thaliana*, *Caenorhabditis elegans*, *Drosophila melanogaster*, *Homo sapiens* and *Saccharomyces cerevisiae*) is chosen. For each clade members of the clade itself are not used as outgroups, for example *Drosophila melanogaster* is not used as an outgroup for the insects. *Strigamia maritima* is additionally added as outgroup for the insect clade to make sure insect specific rearrangements are studied and not general arthropod rearrangements. In a first step all but the longest isoform of each gene is removed from the data set to prevent a bias in event rate detection by their influence on the analysis. Proteomes are annotated with *Pfam* domain models [58] (version 30) using the pfam_scan.pl script (version 1.5) provided by *Pfam*. We used default parameters so that the script applies the thresholds specified in the Pfam database for annotating and filtering of the domains. Consecutive domain repeats in arrangements are collapsed to one instance of the domain (A-B-B-B-C $\rightarrow $ A-B-C), as it has been shown that even between closely related species copy number of repeated domains can vary a lot [59] and also to avoid miscalculations due to split domains caused by annotation/gene model errors.

The phylogenetic tree for the vertebrate clade is taken from ensembl [57]. The fungi tree is built using *NCBI Taxonomy database* [60] and *Superfamily* [2] as basis and resolving unknown branches from literature [61, 62]. The insect tree is built according to the *NCBI Taxonomy database*, while multifurcating branches of the genera Papilio, Apis, Bombus and Dufourea are transformed to bifurcating solutions according to literature [63–66]. Plant phylogenies are initially inferred using *NCBI Taxonomy* and refined using literature [67–69]. Next to the quality criterion mentioned above the resolvability of the phylogenetic relationship to other species was the second crucial criterion for the sampling process. The effect of subsampling replicates on the analysis is discussed based on a jackknife test.

### Reconstruction of ancestral domain arrangements

The reconstruction of ancestral domain arrangements and calculation of rates of domain rearrangement events is carried out using the in-house developed program ’DomRates’ (http://domainworld.uni-muenster.de/programs/domrates/).

**Fig. 3:**
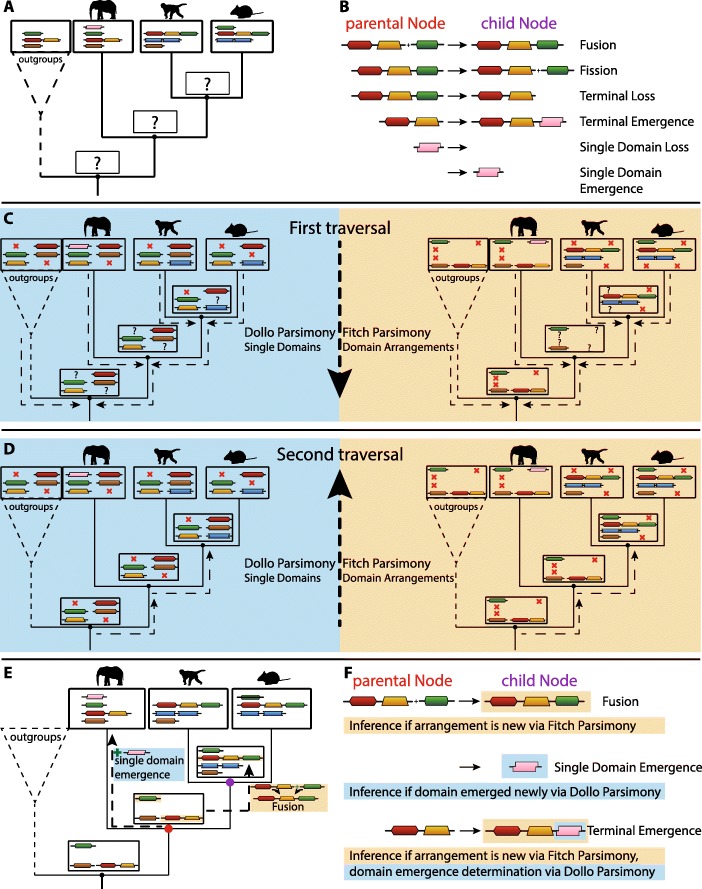
Reconstruction of ancestral domain content and rearrangement events. Given a known phylogeny and domain annotations of all included species (**a**), it becomes possible to infer six event types leading to new domain contents over time (**b**). First, the ancestral domain content of all inner nodes is inferred by two different parsimony approaches: for all single domains using a Dollo parsimony approach (light blue background), and for all arrangements, using a Fitch parsimony approach (light orange background). In a first traversal from the leaves to the root of the tree, all inner node states are annotated as present, absent or unknown according to the regarding parsimony rules (**c**) (see Additional file 1). In a second traversal from the root to the leaves, the unknown states at the root are first resolved according to the parsimony rules (see Additional file 1) and subsequently all following unknown states set to the parental state (**d**). In the reconstructed tree it becomes possible to infer the different event types at any node by comparison with the parental node (**e**). In this way emergences/losses of domains are inferred from the Dollo tree, while arrangements are inferred from the Fitch tree (**f**)

Reconstruction of ancestral states of domains and domain arrangements is based on a parsimony principle. While single domain presence/absence states are usually better modelled by a Dollo parsimony, multi-domain arrangements with their modular nature are better modelled by a Fitch parsimony. The assumption underlying the use of Dollo parsimony is that novel domains are gained only once [16], while arrangements can be formed and broken several times. For this reason, ’DomRates’ reconstructs the ancestral states of the whole tree twice: First with Fitch parsimony for all domain arrangements (including single domain arrangements) and a second time with Dollo parsimony for all single domains included in any arrangement (see Fig. 3). The inferred single domain states with Dollo parsimony are used to verify all terminal emergence events and single domain loss/emergence events found by the Fitch parsimony reconstruction.

The copy number of certain domain arrangements is not considered in DomRates, which means only the presence/absence of a given arrangement is reconstructed and taken into account, but not the number of appearances in the proteome. This means that emergence and loss are seen from a functional perspective in this study based on if an arrangement is available in the functional repertoire of a proteome. Expansions and contractions of the same arrangement regarding the numbers of its copies are not described as emergence or loss.

### Terms and definitions - event and solution types

Since previous research in the field of protein domains focused mainly either on emergence and loss of single domains or on the evolutionary history of whole arrangements, sometimes postulating concepts such as recombination or domain-shuffling, it is necessary to specify the rearrangement events considered in this study (see Fig. 3b). In fact, just four biological events can explain the formation of virtually all domain arrangements: *fusion* of existing (ancestral) arrangements (also of single domain proteins which amounts to gene fusion), *fission* of existing (ancestral) domain arrangements, *loss* of one or more domains (i.e. there are no traces left as the underlying DNA sequence is for example no longer transcribed) and *emergence* of one domain. The latter two biological events of *loss* and *emergence* can be divided into two different conceptual ones each. We distinguish in our study terminal loss/emergence and single domain loss/emergence, which can be both explained by the underlying mechanisms for loss and emergence. Terminal events describe the loss or emergence of domains at the ends of arrangements, while single domain events describe the complete loss or the first emergence of a single domain as a discrete arrangement. Terminal loss allows for more than one domain to be lost in contrast to just one domain considered for terminal emergence, since terminal loss can easily be caused by an introduced stop codon, which affects dependent on the position all following domains in the protein and not just the next or last domain. With this conceptual differentiation we make it possible to combine the two different approaches of previous studies (loss and emergence of single domains vs. reshuffling of domain arrangements).

It is important to note that all mutational events described here are defined purely on a domain level. On a DNA level different molecular mechanisms and mutations can lead to the same mutational event described here (e.g. fusion of two arrangements by fusion of neighboring genes through stop codon loss or through transposition of a second gene through mobile elements). For this reason we just define events we can infer explicitly on a domain level, while other potential molecular mechanisms leading to additional (less common) mutational events are not considered. An example for this would be the insertion of a domain/arrangement in the middle of an existing domain arrangement, which can happen through crossing over or transposition through smaller mobile elements, but cannot be distinguished on a domain level between insertion in the middle of an arrangement or two subsequent fusion events of independent arrangements. The possibility of multi step events or multiple possible solutions makes the definition of different solution types necessary.

One can differentiate between four different solution types (see Additional file 2): exact solution, non-ambiguous solution, and ambiguous solution can all be explained by one instance of the single step event types above, while a complex solution can only be explained by a chain of the above mentioned events. Exact solutions represent new arrangements that can be explained by a single event and just this one solution exists. In contrast, non-ambiguous solutions describe the case that a new arrangement can just be explained by one out of several single events, all of the same type. Ambiguous solutions involve more than one event type as a possible explanation for a new arrangement. If there does not exist a solution in a single step, it is defined as a complex solution.

### Domain rearrangement rates calculation

For the rate determination only exact and non-ambiguous solutions are considered, ambiguous and complex solutions are ignored. To avoid bias introduced by outgroup-specific arrangements, we exclude the nodes of the outgroup, the root of the complete tree and the root of each clade (first node after root) from the rate calculation. A jackknife test with 100 repetitions is carried out by randomly removing 3 species from every clade and rerunning DomRates on the altered phylogeny to ensure robustness of the found rates and to identify possible sampling biases within clades. Means and standard deviation for every event type frequency in the jackknife test are shown in Additional file 4.

### Enriched gene ontology terms

A Gene Ontology (GO) term enrichment is carried out with *topGO* package [70] in R. The GO universe is composed of all domain arrangements that are present in all species in a clade as well as the reconstructed domain arrangements set in the ancestral nodes. Domains in new domain arrangements that can be explained by an exact or non-ambiguous solution are annotated with the ’pfam2go’ mapping of *Pfam* domains to GO terms [71]. The enrichment analysis is done using the ontologies of ’Molecular function’ and ’Biological process’ and *topGO*s ’weight01’ algorithm. Significantly enriched (*P*-value ≤0.05) GO terms are visualized as tag clouds.

## Supplementary information

**Additional file 1** Rules of inference for both parsimony approaches. The middle panel shows which two parental states (present, absent or unknown) for a domain or arrangement lead to which inference in the child node according to Dollo parsimony (left) or Fitch parsimony (right). The last line shows to what state an unknown state at the root is resolved.

**Additional file 2** Solution types. There are four different solution types by which a new arrangement can be explained. Exact and non-ambiguous solutions involve each just one event type (see Fig. 3b) and are called unique solutions. Ambiguous and complex solutions cannot be explained by a single event type and are called manifold solutions. Just unique solutions are considered for the rate calculation in this study.

**Additional file 3** Total number of events per solution type for all five clades.

**Additional file 4** Jackknife test. Mean and standard deviation for all event type frequencies of a jackknife test with 100 replicates. For the jackknife test 3 species per clade were randomly removed and the resulting phylogeny tested with DomRates (100 repetitions).

**Additional file 5** Number of rearrangement events across the insect phylogeny. Digit representation of the total number of rearrangement events at a specific node is indicated next to the pie chart. For details on ‘Outgroups’ see Methods. Significant GO terms in gained domain arrangements are shown in a tag cloud (box). GO terms that might point to insect specific evolution are: chitin metabolic process, sensory perception of taste.

**Additional file 6** Number of rearrangement events across the vertebrate phylogeny. Digit representation of the total number of rearrangement events at a specific node is indicated next to the pie chart. For details on ‘Outgroups’ see Methods. Significant GO terms in gained domain arrangements are shown in a tag cloud (box). GO terms related to vertebrate evolution are strongly associated with regulation and signal transduction.

**Additional file 7** Number of rearrangement events across the fungi phylogeny. Digit representation of the total number of rearrangement events at a specific node is indicated next to the pie chart. For details on ’Outgroups’ see Methods. Significant GO terms in gained domain arrangements are shown in a tag cloud (box).

**Additional file 8** Number of rearrangement events across the monocot phylogeny. Digit representation of the total number of rearrangement events at a specific node is indicated next to the pie chart. For details on ’Outgroups’ see Methods. Significant GO terms in gained domain arrangements are shown in a tag cloud (box). GO terms that might point to monocot specific evolution are: ’recognition of pollen’.

**Additional file 9** Domain arrangement sizes. The size represents the number of domains an arrangement consists of, while the fraction relates to all discriminative domain arrangements in total for the specific clade. The total number of different arrangements considered in the data sets was 22199 (vertebrates), 22346 (insects), 10030 (fungi), 15565 (monocots) and 12097 (eudicots).

**Additional file 10** GO term enrichment analysis. Tag cloud for all events at the root of mammals in the vertebrate tree.

**Additional file 11** List of all species included in this study. Furthermore, for each species the related DOGMA completeness score of their proteome and version of the used genome assembly is shown.

## Data Availability

The developed software DomRates is available via the project homepage http://domainworld.uni-muenster.de/programs/domrates/ and with full source code on our gitlab https://ebbgit.uni-muenster.de/domainWorld/DomRates/. Furthermore, an archived version of the source code with all data related to this study (DomRates result files, GOterm analysis, jackknife test) is available via https://doi.org/10.5281/zenodo.2630419. DomRates is implemented in C++ and platform independent, published under the free GNU GPL licence version 3. For further information please check the UserManual on the website, where you can find also tutorials and example data sets to test the software.

## Abbreviations

EGF: Epidermal growth factor
GO: Gene Ontology
IIS: Insulin and insulin-like signalling
KRTAP: Keratin-associated protein
KRTDAP: Keratinocyte differentiation-associated protein
