# Supplementary figures and images for: The modular nature of protein evolution: domain rearrangement rates across eukaryotic life
Source: BMC Evol Biol. 2020 Feb 14;20:30. doi: 10.1186/s12862-020-1591-0 (PMC7023805; doi:10.1186/s12862-020-1591-0)

## Dollo Parsimony

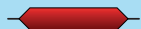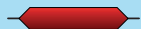

×

?

?

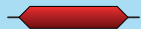

×

## child node states

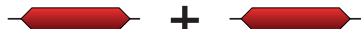

+

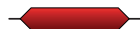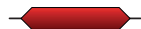

+

?

×

+

×

?

+

×

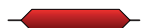

+

×

?

+

?

? at root

## Fitch Parsimony

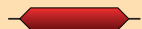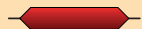

×

×

?

?

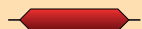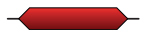

present

×

absent

?

unknown

Supplement: Supplementary file 1 — Additional file 1 Rules of inference for both parsimony approaches. The middle panel shows which two parental states (present, absent or unknown) for a domain or arrangement lead to which inference in the child node according to Dollo parsimony (left) or Fitch parsimony (right). The last line shows to what state an unknown state at the root is resolved. [file 12862_2020_1591_MOESM1_ESM.pdf]

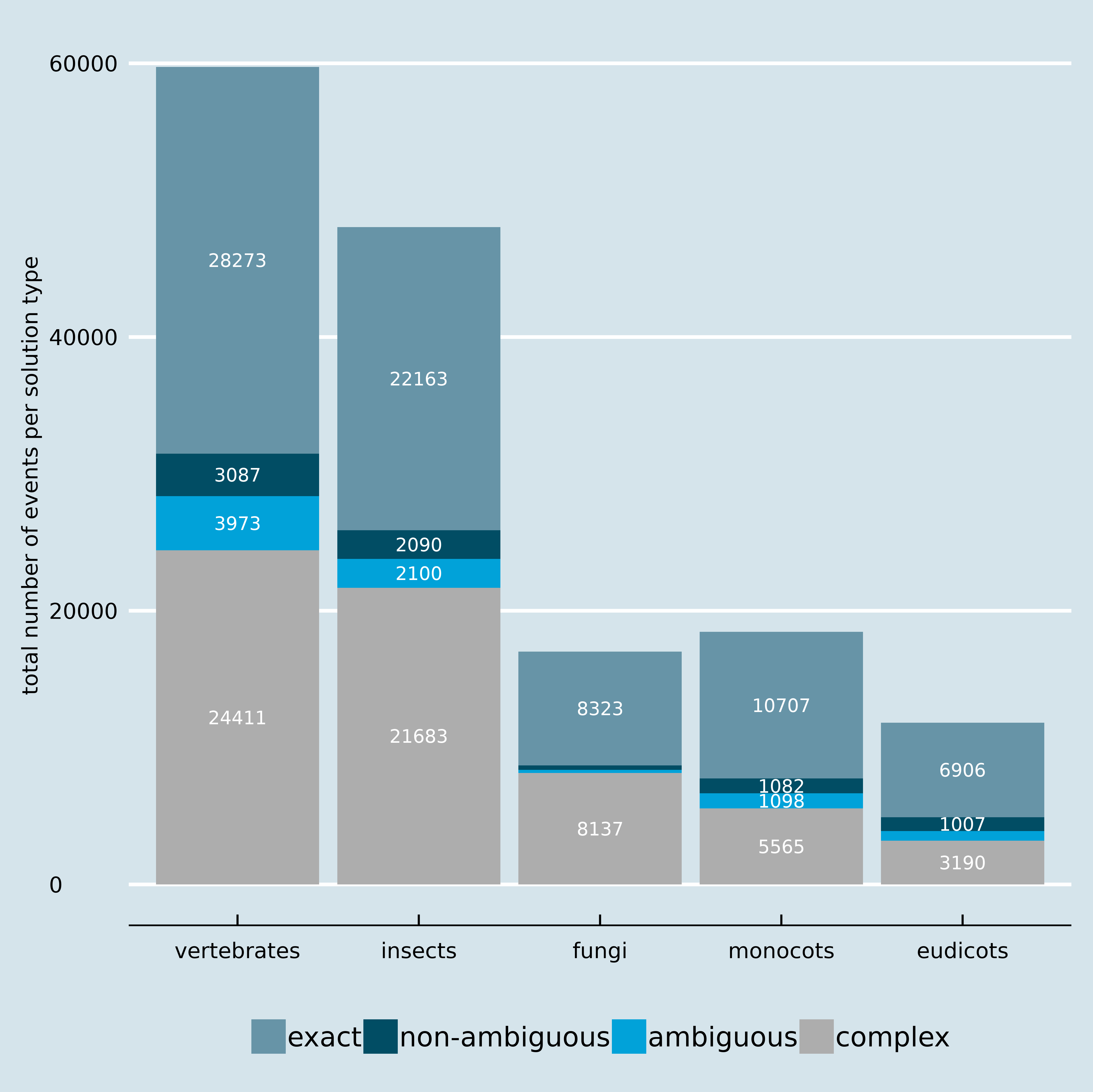

Supplement: Supplementary file 3 — Additional file 3 Total number of events per solution type for all five clades. [file 12862_2020_1591_MOESM3_ESM.png]

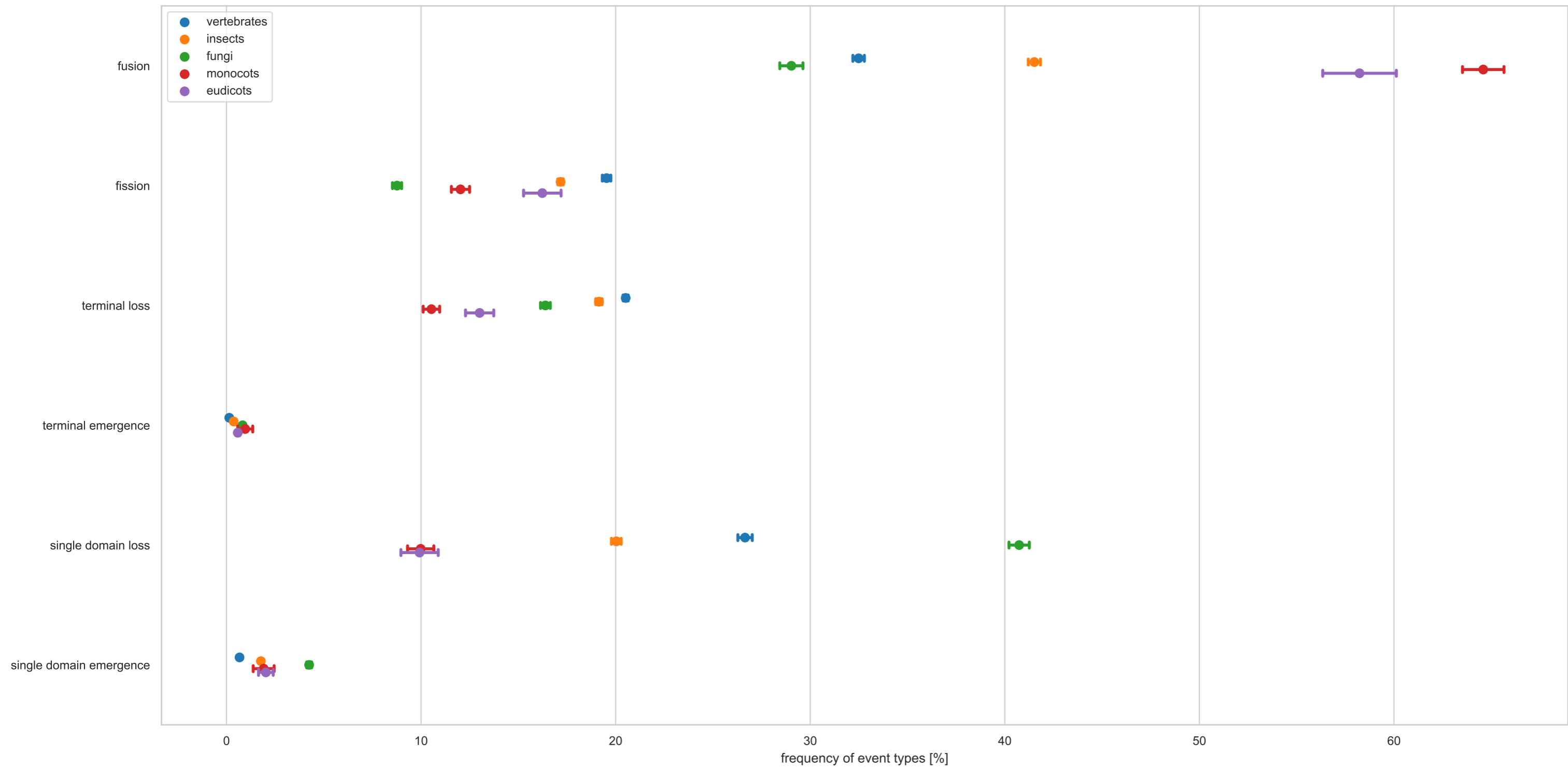

Supplement: Supplementary file 4 — Additional file 4 Jackknife test. Mean and standard deviation for all event type frequencies of a jackknife test with 100 replicates. For the jackknife test 3 species per clade were randomly removed and the resulting phylogeny tested with DomRates (100 repetitions). [file 12862_2020_1591_MOESM4_ESM.pdf]

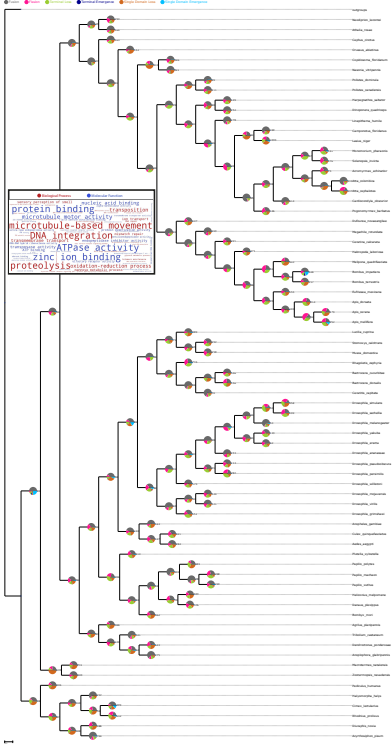

Supplement: Supplementary file 5 — Additional file 5 Number of rearrangement events across the insect phylogeny. Digit representation of the total number of rearrangement events at a specific node is indicated next to the pie chart. For details on ‘Outgroups’ see Methods. Significant GO terms in gained domain arrangements are shown in a tag cloud (box). GO terms that might point to insect specific evolution are: chitin metabolic process, sensory perception of taste. [file 12862_2020_1591_MOESM5_ESM.pdf]

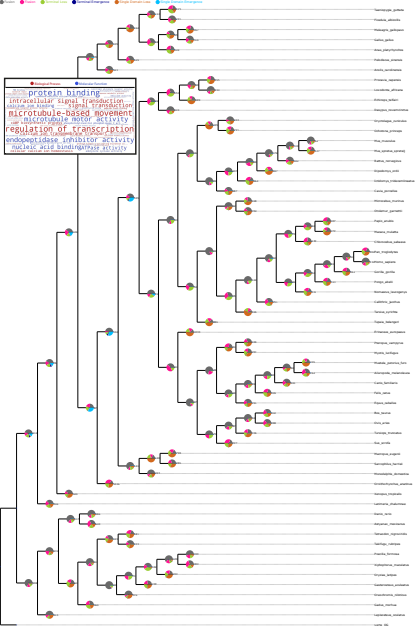

Supplement: Supplementary file 6 — Additional file 6 Number of rearrangement events across the vertebrate phylogeny. Digit representation of the total number of rearrangement events at a specific node is indicated next to the pie chart. For details on ‘Outgroups’ see Methods. Significant GO terms in gained domain arrangements are shown in a tag cloud (box). GO terms related to vertebrate evolution are strongly associated with regulation and signal transduction. [file 12862_2020_1591_MOESM6_ESM.pdf]

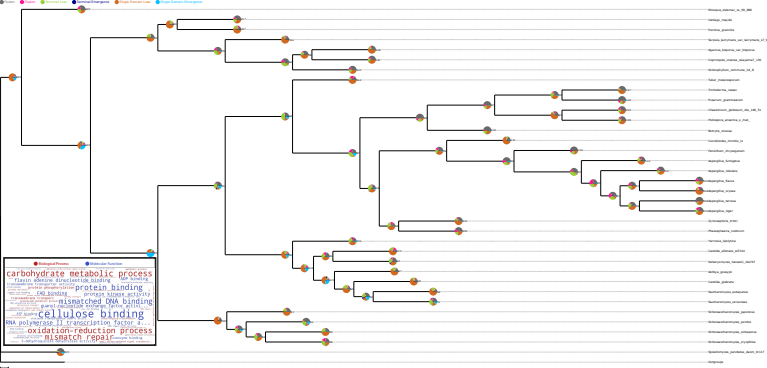

Supplement: Supplementary file 7 — Additional file 7 Number of rearrangement events across the fungi phylogeny. Digit representation of the total number of rearrangement events at a specific node is indicated next to the pie chart. For details on ’Outgroups’ see Methods. Significant GO terms in gained domain arrangements are shown in a tag cloud (box). [file 12862_2020_1591_MOESM7_ESM.pdf]

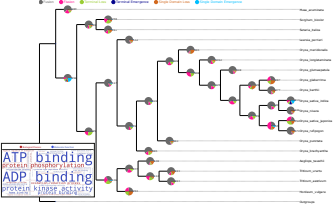

Supplement: Supplementary file 8 — Additional file 8 Number of rearrangement events across the monocot phylogeny. Digit representation of the total number of rearrangement events at a specific node is indicated next to the pie chart. For details on ’Outgroups’ see Methods. Significant GO terms in gained domain arrangements are shown in a tag cloud (box). GO terms that might point to monocot specific evolution are: ’recognition of pollen’. [file 12862_2020_1591_MOESM8_ESM.pdf]

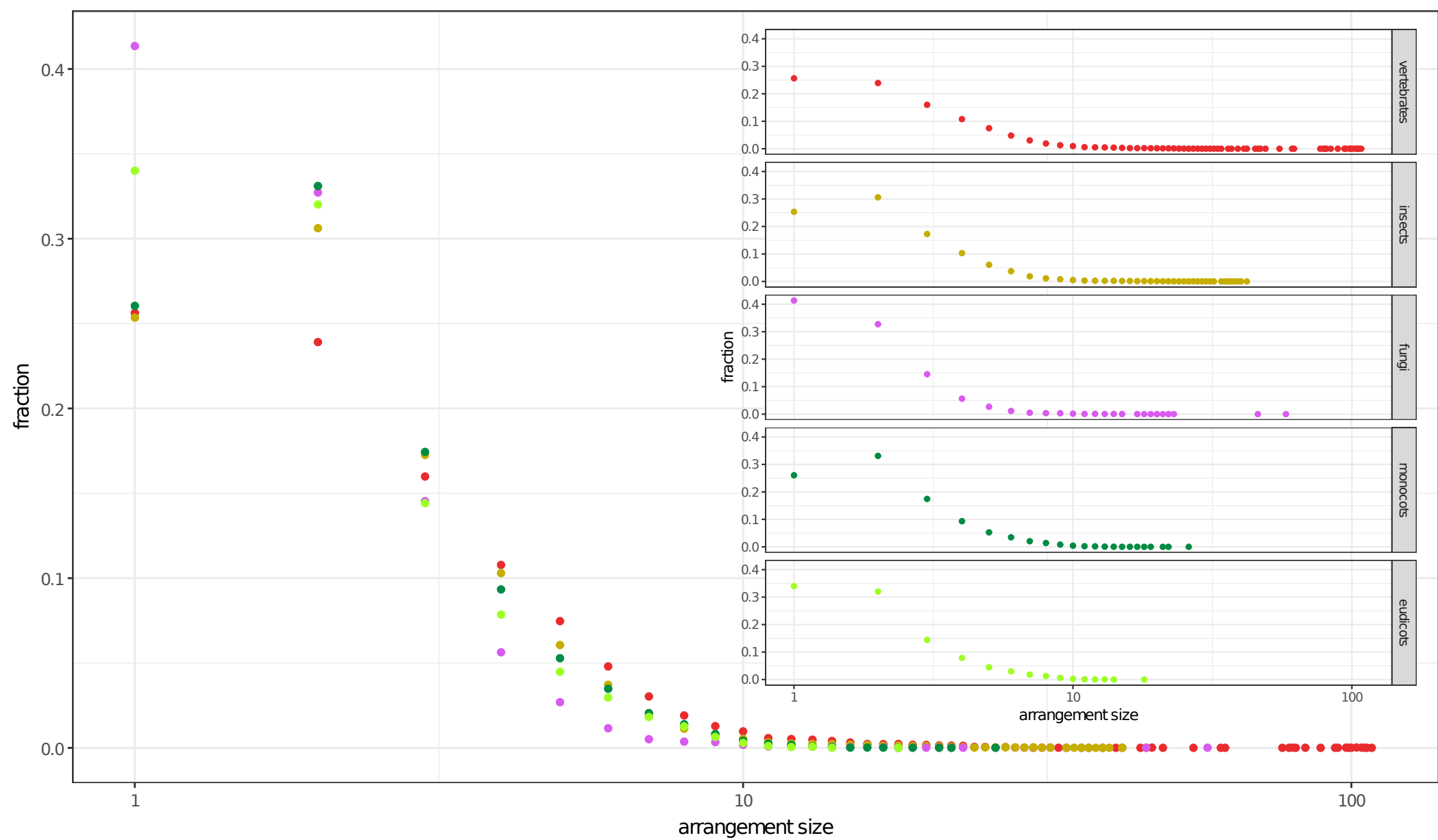

Supplement: Supplementary file 9 — Additional file 9 Domain arrangement sizes. The size represents the number of domains an arrangement consists of, while the fraction relates to all discriminative domain arrangements in total for the specific clade. The total number of different arrangements considered in the data sets was 22199 (vertebrates), 22346 (insects), 10030 (fungi), 15565 (monocots) and 12097 (eudicots). [file 12862_2020_1591_MOESM9_ESM.pdf]
