# Supplementary material for: The modular nature of protein evolution: domain rearrangement rates across eukaryotic life
Source: BMC Evol Biol. 2020 Feb 14;20:30. doi: 10.1186/s12862-020-1591-0 (PMC7023805; doi:10.1186/s12862-020-1591-0)
Supplement: Supplementary file 2 — Additional file 2 Solution types. There are four different solution types by which a new arrangement can be explained. Exact and non-ambiguous solutions involve each just one event type (see Fig. 3b) and are called unique solutions. Ambiguous and complex solutions cannot be explained by a single event type and are called manifold solutions. Just unique solutions are considered for the rate calculation in this study. [file 12862_2020_1591_MOESM2_ESM.pdf]

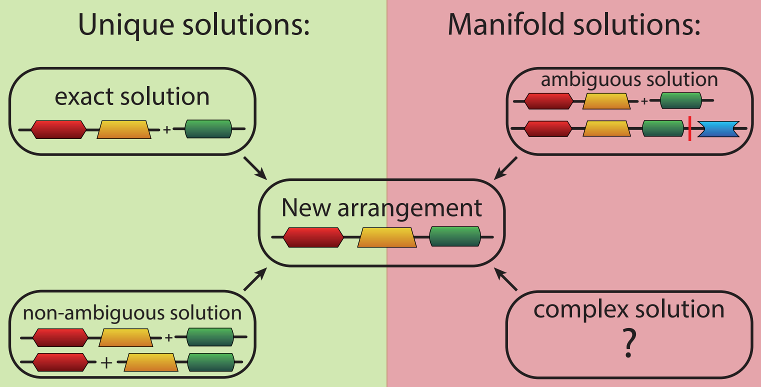
Unique solutions:
Manifold solutions:
exact solution
ambiguous solution
New arrangement
non-ambiguous solution
complex solution
?
