# Supplementary material for: The modular nature of protein evolution: domain rearrangement rates across eukaryotic life
Source: BMC Evol Biol. 2020 Feb 14;20:30. doi: 10.1186/s12862-020-1591-0 (PMC7023805; doi:10.1186/s12862-020-1591-0)
Supplement: Supplementary file 10 — Additional file 10 GO term enrichment analysis. Tag cloud for all events at the root of mammals in the vertebrate tree. [file 12862_2020_1591_MOESM10_ESM.pdf]

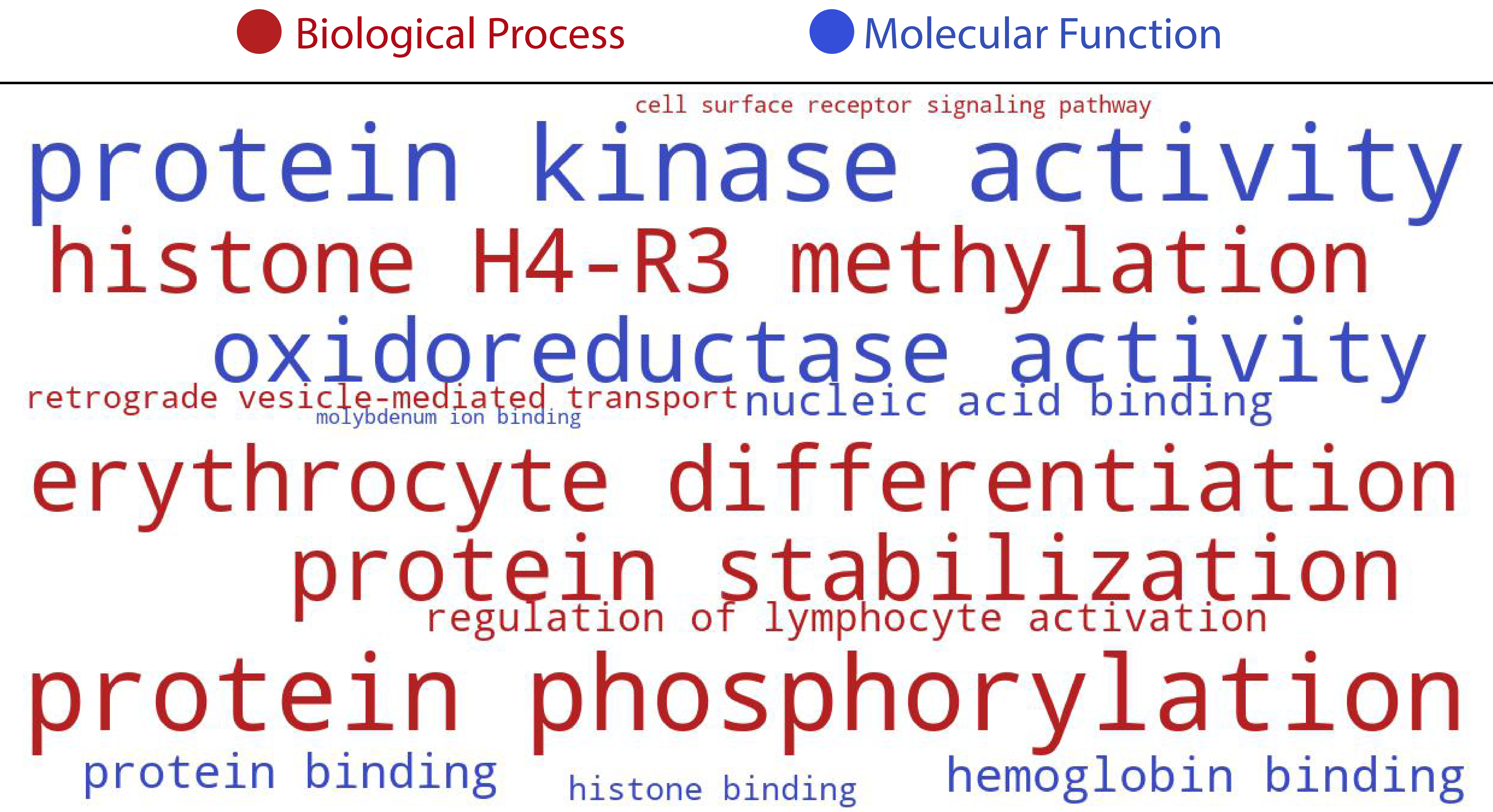

Biological Process
Molecular Function
cell surface receptor signaling pathway
protein kinase activity
histone H4-R3 methylation
oxidoreductase activity
retrograde vesicle-mediated transport
nucleic acid binding
molybdenum ion binding
erythrocyte differentiation
protein stabilization
regulation of lymphocyte activation
protein phosphorylation
protein binding
histone binding
hemoglobin binding
