# Supplementary material for: The modular nature of protein evolution: domain rearrangement rates across eukaryotic life
Source: BMC Evol Biol. 2020 Feb 14;20:30. doi: 10.1186/s12862-020-1591-0 (PMC7023805; doi:10.1186/s12862-020-1591-0)
Supplement: Supplementary file 11 — Additional file 11 List of all species included in this study. Furthermore, for each species the related DOGMA completeness score of their proteome and version of the used genome assembly is shown. [file 12862_2020_1591_MOESM11_ESM.pdf]

**S11 Table**

| Data Set | Species | DOGMA Score [%] | Source - assembly version | Reference [doi] |
|---|---|---|---|---|
| Vertebrates | Ailuropoda melanoleuca | 98,81 | ensemble v86 | 10.1093/database/baw093 |
| Vertebrates | Anas platyrhynchos | 88,35 | ensemble v86 | 10.1093/database/baw093 |
| Vertebrates | Anolis carolinensis | 92,46 | ensemble v86 | 10.1093/database/baw093 |
| Vertebrates | Astyanax mexicanus | 93,18 | ensemble v86 | 10.1093/database/baw093 |
| Vertebrates | Bos taurus | 98,81 | ensemble v86 | 10.1093/database/baw093 |
| Vertebrates | Callithrix jacchus | 98,48 | ensemble v86 | 10.1093/database/baw093 |
| Vertebrates | Canis familiaris | 97,22 | ensemble v86 | 10.1093/database/baw093 |
| Vertebrates | Cavia porcellus | 97,62 | ensemble v86 | 10.1093/database/baw093 |
| Vertebrates | Chlorocebus sabaeus | 97,02 | ensemble v86 | 10.1093/database/baw093 |
| Vertebrates | Danio rerio | 98,21 | ensemble v86 | 10.1093/database/baw093 |
| Vertebrates | Dasypus novemcinctus | 96,36 | ensemble v86 | 10.1093/database/baw093 |
| Vertebrates | Dipodomys ordii | 86,5 | ensemble v86 | 10.1093/database/baw093 |
| Vertebrates | Echinops telfairi | 83,92 | ensemble v86 | 10.1093/database/baw093 |
| Vertebrates | Equus caballus | 97,88 | ensemble v86 | 10.1093/database/baw093 |
| Vertebrates | Erinaceus europaeus | 77,56 | ensemble v86 | 10.1093/database/baw093 |
| Vertebrates | Felis catus | 97,62 | ensemble v86 | 10.1093/database/baw093 |
| Vertebrates | Ficedula albicollis | 90,07 | ensemble v86 | 10.1093/database/baw093 |
| Vertebrates | Gadus morhua | 96,16 | ensemble v86 | 10.1093/database/baw093 |
| Vertebrates | Gallus gallus | 91,66 | ensemble v86 | 10.1093/database/baw093 |
| Vertebrates | Gasterosteus aculeatus | 97,02 | ensemble v86 | 10.1093/database/baw093 |
| Vertebrates | Gorilla gorilla | 96,69 | ensemble v86 | 10.1093/database/baw093 |
| Vertebrates | Homo sapiens | 100 | ensemble v86 | 10.1093/database/baw093 |
| Vertebrates | Ictidomys tridecemlineatus | 90,27 | ensemble v86 | 10.1093/database/baw093 |
| Vertebrates | Latimeria chalumnae | 93,51 | ensemble v86 | 10.1093/database/baw093 |
| Vertebrates | Lepisosteus oculatus | 94,37 | ensemble v86 | 10.1093/database/baw093 |
| Vertebrates | Loxodonta africana | 98,01 | ensemble v86 | 10.1093/database/baw093 |
| Vertebrates | Macaca mulatta | 96,29 | ensemble v86 | 10.1093/database/baw093 |
| Vertebrates | Macropus eugenii | 83,65 | ensemble v86 | 10.1093/database/baw093 |
| Vertebrates | Meleagris gallopavo | 82,86 | ensemble v86 | 10.1093/database/baw093 |
| Vertebrates | Microcebus murinus | 94,11 | ensemble v86 | 10.1093/database/baw093 |
| Vertebrates | Monodelphis domestica | 96,89 | ensemble v86 | 10.1093/database/baw093 |
| Vertebrates | Mus musculus | 99,87 | ensemble v86 | 10.1093/database/baw093 |
| Vertebrates | Mus spretus | 98,41 | ensemble v86 | 10.1093/database/baw093 |
| Vertebrates | Mustela putorius | 95,63 | ensemble v86 | 10.1093/database/baw093 |
| Vertebrates | Myotis lucifugus | 96,56 | ensemble v86 | 10.1093/database/baw093 |
| Vertebrates | Nomascus leucogenys | 95,43 | ensemble v86 | 10.1093/database/baw093 |
| Vertebrates | Ochotona princeps | 85,77 | ensemble v86 | 10.1093/database/baw093 |
| Vertebrates | Oreochromis niloticus | 95,63 | ensemble v86 | 10.1093/database/baw093 |
| Vertebrates | Ornithorhynchus anatinus | 83,45 | ensemble v86 | 10.1093/database/baw093 |
| Vertebrates | Oryctolagus cuniculus | 93,85 | ensemble v86 | 10.1093/database/baw093 |
| Vertebrates | Oryzias latipes | 92,98 | ensemble v86 | 10.1093/database/baw093 |
| Vertebrates | Otolemur garnettii | 95,43 | ensemble v86 | 10.1093/database/baw093 |
| Vertebrates | Ovis aries | 97,95 | ensemble v86 | 10.1093/database/baw093 |
| Vertebrates | Pan troglodytes | 95,43 | ensemble v86 | 10.1093/database/baw093 |
| Vertebrates | Papio anubis | 95,63 | ensemble v86 | 10.1093/database/baw093 |
| Vertebrates | Pelodiscus sinensis | 88,62 | ensemble v86 | 10.1093/database/baw093 |
| Vertebrates | Poecilia formosa | 98,48 | ensemble v86 | 10.1093/database/baw093 |
| Vertebrates | Pongo abelii | 95,5 | ensemble v86 | 10.1093/database/baw093 |
| Vertebrates | Procavia capensis | 85,97 | ensemble v86 | 10.1093/database/baw093 |
| Vertebrates | Pteropus vampyrus | 94,24 | ensemble v86 | 10.1093/database/baw093 |
| Vertebrates | Rattus norvegicus | 98,54 | ensemble v86 | 10.1093/database/baw093 |

| | | | | |
|---|---|---|---|---|
| Vertebrates | Sarcophilus harrisii | 90,14 | ensemble v86 | 10.1093/database/baw093 |
| Vertebrates | Sus scrofa | 89,15 | ensemble v86 | 10.1093/database/baw093 |
| Vertebrates | Taeniopygia guttata | 87,29 | ensemble v86 | 10.1093/database/baw093 |
| Vertebrates | Takifugu rubripes | 95,5 | ensemble v86 | 10.1093/database/baw093 |
| Vertebrates | Tarsius syrichta | 78,89 | ensemble v86 | 10.1093/database/baw093 |
| Vertebrates | Tetraodon nigroviridis | 93,98 | ensemble v86 | 10.1093/database/baw093 |
| Vertebrates | Tupaia belangeri | 81,4 | ensemble v86 | 10.1093/database/baw093 |
| Vertebrates | Tursiops truncatus | 95,37 | ensemble v86 | 10.1093/database/baw093 |
| Vertebrates | Xenopus tropicalis | 94,97 | ensemble v86 | 10.1093/database/baw093 |
| Vertebrates | Xiphophorus maculatus | 96,76 | ensemble v86 | 10.1093/database/baw093 |
| Insects | Acromyrmex echinatior | 96,6 | NCBI - v3.9 | 10.1101/gr.121392.111 |
| Insects | Acyrthosiphon pisum | 89,25 | NCBI - v2.0 | 10.1371/journal.pbio.1000313 |
| Insects | Aedes aegypti | 93,74 | NCBI - AaegL3 | 10.1126/science.1138878 |
| Insects | Agrilus planipennis | 91,26 | NCBI - v1.0 | 10.1093/jhered/est050 |
| Insects | Anopheles gambiae | 99,95 | NCBI - AgamP3 | 10.1186/gb-2007-8-1-r5 |
| Insects | Anoplophora glabripennis | 96,62 | NCBI - v1.0 | 10.1093/jhered/est050 |
| Insects | Apis cerana | 94,53 | NCBI - v2.0 | 10.1186/1471-2164-16-1 |
| Insects | Apis dorsata | 97,38 | NCBI - v1.3 | |
| Insects | Apis mellifera | 96,11 | NCBI - v4.5 | 10.1016/j.gdata.2015.01.011 |
| Insects | Athalia rosae | 97,69 | NCBI - v1.0 | 10.1093/jhered/est050 |
| Insects | Atta cephalotes | 94,88 | NCBI - v1.0 | 10.1371/journal.pgen.1002007 |
| Insects | Atta colombica | 96,71 | NCBI - v1.0 | |
| Insects | Bactrocera cucurbitae | 96,71 | NCBI - ASM80634v1 | |
| Insects | Bactrocera dorsalis | 94,97 | NCBI - ASM78921v2 | |
| Insects | Bombus impatiens | 100 | NCBI - v2.0 | 10.1186/s13059-015-0623-3 |
| Insects | Bombus terrestris | 97,98 | NCBI - v1.0 | 10.1186/s13059-015-0623-4 |
| Insects | Bombyx mori | 80,99 | NCBI - ASM15162v1 | 10.1016/j.ibmb.2008.11.004 |
| Insects | Camponotus floridanus | 96,59 | NCBI - v1.0 | 10.1126/science.1192428 |
| Insects | Cardiocondyla obscurior | 95,27 | hymenopteragenome.org - v1.4 | 10.1093/nar/gkv120810 |
| Insects | Cephus cinctus | 97,9 | NCBI - v1.0 | |
| Insects | Ceratina calcarata | 95,95 | NCBI - ASM165200v1 | |
| Insects | Ceratitis capitata | 96,73 | NCBI - v1.1 | 10.1093/jhered/est050 |
| Insects | Cimex lectularius | 93,33 | NCBI - v1.0 | 10.1038/ncomms10164 |
| Insects | Copidosoma floridanum | 91,53 | NCBI - v1.0 | 10.1093/jhered/est050 |
| Insects | Culex quinquefasciatus | 89,32 | NCBI - v1.0 | |
| Insects | Danaus plexippus | 85,67 | NCBI - v1.0 | 10.1016/j.cell.2011.09.052 |
| Insects | Dendroctonus ponderosae | 86,63 | NCBI - v1.0 | 10.1186/gb-2013-14-3-r27 |
| Insects | Dinoponera quadriceps | 96,82 | NCBI - ASM131382v1 | |
| Insects | Diuraphis noxia | 84,62 | NCBI - v1.0 | 10.1186/s12864-015-1525-1 |
| Insects | Drosophila ananassae | 96,86 | NCBI - v1.0 | 10.1038/nature06341 |
| Insects | Drosophila erecta | 96,93 | NCBI - v1.0 | 10.1038/nature06341 |
| Insects | Drosophila grimshawi | 96,65 | NCBI - v1.0 | 10.1038/nature06341 |
| Insects | Drosophila melanogaster | 99,98 | NCBI - v6.0 | 10.1101/gr.185579.114 |
| Insects | Drosophila mojavensis | 95,16 | NCBI - v1.0 | 10.1038/nature06341 |
| Insects | Drosophila persimilis | 89,06 | NCBI - v1.0 | 10.1038/nature06341 |
| Insects | Drosophila pseudoobscura | 96,22 | NCBI - v3.0 | 10.1101/gr.3059305 |
| Insects | Drosophila sechellia | 91,67 | NCBI - v1.0 | 10.1038/nature06341 |
| Insects | Drosophila simulans | 81,01 | NCBI - ASM75419v2 | 10.1038/nature06341 |
| Insects | Drosophila virilis | 95,81 | NCBI - v1.0 | 10.1038/nature06341 |
| Insects | Drosophila willistoni | 97,09 | NCBI - v1.0 | 10.1038/nature06341 |
| Insects | Drosophila yakuba | 96,93 | NCBI - v1.0 | 10.1038/nature06341 |
| Insects | Dufourea novaeangliae | 96,7 | NCBI - ASM127255v1 | |
| Insects | Eufriesea mexicana | 96,82 | NCBI - ASM148370v1 | |
| Insects | Habropoda laboriosa | 96,82 | NCBI - ASM126327v1 | |

| | | | | |
|---|---|---|---|---|
| Insects | Halyomorpha halys | 92,92 | NCBI - v1.0 | |
| Insects | Harpegnathos saltator | 96,86 | NCBI - v1.0 | 10.1126/science.1192428 |
| Insects | Heliconius melpomene | 75,93 | ensemble metazoa v34 | 10.1093/database/baw093 |
| Insects | Lasius niger | 77,05 | NCBI - ASM104565v1 | |
| Insects | Linepithema humile | 97,52 | NCBI - Lhum_UMD_V04 | 10.1073/pnas.1008617108 |
| Insects | Lucilia cuprina | 85,87 | NCBI - ASM118794v1 | 10.1038/ncomms8344 |
| Insects | Macrotermes natalensis | 80,64 | ftp://climb.genomics.cn/... pub/10.5524/100001_101000/100057 - v1.2 | 10.5524/100057 |
| Insects | Megachile rotundata | 97,53 | NCBI - v1.0 | |
| Insects | Melipona quadrifasciata | 86,3 | NCBI - ASM127656v1 | |
| Insects | Monomorium pharaonis | 96,79 | NCBI - v2.0 | |
| Insects | Musca domestica | 95,88 | NCBI - v2.0.2 | 10.1186/s13059-014-0466-3 |
| Insects | Nasonia vitripennis | 83,83 | NCBI - v2.1 | 10.1126/science.1178028 |
| Insects | Neodiprion lecontei | 97,63 | NCBI - v1.0 | |
| Insects | Orussus abietinus | 96,13 | NCBI - v1.0 | |
| Insects | Papilio machaon | 89,38 | NCBI - v1.0 | |
| Insects | Papilio polytes | 84,67 | NCBI - v1.0 | |
| Insects | Papilio xuthus | 93,36 | NCBI - v1.0 | |
| Insects | Pediculus humanus | 91,18 | NCBI - v1.0 | 10.1073/pnas.1003379107 |
| Insects | Plutella xylostella | 85,68 | NCBI - v1.1 | 10.1038/ng.2524 |
| Insects | Pogonomyrmex barbatus | 95,34 | NCBI - Pbar_UMD_V03 | 10.1073/pnas.1007901108 |
| Insects | Polistes canadensis | 96,54 | NCBI - ASM131383v1 | |
| Insects | Polistes dominula | 97,09 | NCBI - v1.2 | |
| Insects | Rhagoletis zephyria | 96,41 | NCBI - v1.0 | |
| Insects | Rhodnius prolixus | 80,67 | ensemble metazoa v34 | 10.1093/database/baw093 |
| Insects | Solenopsis invicta | 94,83 | NCBI - Si_gnG | 10.1073/pnas.1009690108 |
| Insects | Stomoxys calcitrans | 96,19 | NCBI - v1.0.1 | |
| Insects | Tribolium castaneum | 100 | NCBI - v5.2 | 10.1038/nature06784 |
| Insects | Zootermopsis nevadensis | 84,62 | NCBI - v1.0 | 10.1038/ncomms4636 |
| Fungi | Rhizopus delemar ra 99 880 | 88.68 | ensemble fungi v33 | 10.1093/nar/gkv1209 |
| Fungi | Ustilago maydis | 92.85 | ensemble fungi v33 | 10.1093/nar/gkv1209 |
| Fungi | Puccinia graminis | 89.54 | ensemble fungi v33 | 10.1093/nar/gkv1209 |
| Fungi | Serpula lacrymans var lacrymans s7 9 | 83.12 | ensemble fungi v33 | 10.1093/nar/gkv1209 |
| Fungi | Agaricus bisporus var bisporus | 89.21 | ensemble fungi v33 | 10.1093/nar/gkv1209 |
| Fungi | Coprinopsis cinerea okayama7 130 | 92.32 | ensemble fungi v33 | 10.1093/nar/gkv1209 |
| Fungi | Schizophyllum commune h4 8 | 91.20 | ensemble fungi v33 | 10.1093/nar/gkv1209 |
| Fungi | Tuber melanosporum | 89.94 | ensemble fungi v33 | 10.1093/nar/gkv1209 |
| Fungi | Trichoderma reesei | 93.85 | ensemble fungi v33 | 10.1093/nar/gkv1209 |
| Fungi | Fusarium graminearum | 95.10 | ensemble fungi v33 | 10.1093/nar/gkv1209 |
| Fungi | Chaetomium globosum cbs 148 51 | 88.55 | ensemble fungi v33 | 10.1093/nar/gkv1209 |
| Fungi | Podospora anserina s mat | 92.19 | ensemble fungi v33 | 10.1093/nar/gkv1209 |
| Fungi | Botrytis cinerea | 95.63 | ensemble fungi v33 | 10.1093/nar/gkv1209 |
| Fungi | Coccidioides immitis rs | 94.64 | ensemble fungi v33 | 10.1093/nar/gkv1209 |
| Fungi | Penicillium chrysogenum | 94.84 | ensemble fungi v33 | 10.1093/nar/gkv1209 |
| Fungi | Aspergillus fumigatus | 93.65 | ensemble fungi v33 | 10.1093/nar/gkv1209 |
| Fungi | Aspergillus nidulans | 91.40 | ensemble fungi v33 | 10.1093/nar/gkv1209 |
| Fungi | Aspergillus flavus | 90.21 | ensemble fungi v33 | 10.1093/nar/gkv1209 |
| Fungi | Aspergillus oryzae | 89.21 | ensemble fungi v33 | 10.1093/nar/gkv1209 |

| | | | | |
|---|---|---|---|---|
| Fungi | Aspergillus terreus | 89.21 | ensemble fungi v33 | 10.1093/nar/gkv1209 |
| Fungi | Aspergillus niger | 90.60 | ensemble fungi v33 | 10.1093/nar/gkv1209 |
| Fungi | Zymoseptoria tritici | 92.19 | ensemble fungi v33 | 10.1093/nar/gkv1209 |
| Fungi | Phaeosphaeria nodorum | 91.53 | ensemble fungi v33 | 10.1093/nar/gkv1209 |
| Fungi | Yarrowia lipolytica | 95.63 | ensemble fungi v33 | 10.1093/nar/gkv1209 |
| Fungi | Candida albicans sc5314 | 92.85 | ensemble fungi v33 | 10.1093/nar/gkv1209 |
| Fungi | Debaryomyces hansenii cbs767 | 95.70 | ensemble fungi v33 | 10.1093/nar/gkv1209 |
| Fungi | Ashbya gossypii | 96.23 | ensemble fungi v33 | 10.1093/nar/gkv1209 |
| Fungi | Candida glabrata | 97.62 | ensemble fungi v33 | 10.1093/nar/gkv1209 |
| Fungi | Saccharomyces eubayanus | 96.69 | ensemble fungi v33 | 10.1093/nar/gkv1209 |
| Fungi | Saccharomyces cerevisiae | 100.00 | ensemble fungi v33 | 10.1093/nar/gkv1209 |
| Fungi | Schizosaccharomyces japonicus | 92.79 | ensemble fungi v33 | 10.1093/nar/gkv1209 |
| Fungi | Schizosaccharomyces pombe | 94.18 | ensemble fungi v33 | 10.1093/nar/gkv1209 |
| Fungi | Schizosaccharomyces octosporus | 93.98 | ensemble fungi v33 | 10.1093/nar/gkv1209 |
| Fungi | Schizosaccharomyces cryophilus | 93.58 | ensemble fungi v33 | 10.1093/nar/gkv1209 |
| Fungi | Spizellomyces punctatus daom br117 | 92.92 | ensemble fungi v33 | 10.1093/nar/gkv1209 |
| Dicots | Arabidopsis lyrata | 98.15 | ensemble plants v33 | 10.1093/nar/gkv1209 |
| Dicots | Arabidopsis thaliana | 100.00 | ensemble plants v33 | 10.1093/nar/gkv1209 |
| Dicots | Beta vulgaris | 97.15 | ensemble plants v33 | 10.1093/nar/gkv1209 |
| Dicots | Brassica napus | 98.87 | ensemble plants v33 | 10.1093/nar/gkv1209 |
| Dicots | Brassica oleracea | 98.01 | ensemble plants v33 | 10.1093/nar/gkv1209 |
| Dicots | Brassica rapa | 98.15 | ensemble plants v33 | 10.1093/nar/gkv1209 |
| Dicots | Carica papaya | 83.72 | Cpapaya_113 | 10.1038/nature06856 |
| Dicots | Glycine max | 98.94 | ensemble plants v33 | 10.1093/nar/gkv1209 |
| Dicots | Medicago truncatula | 98.15 | ensemble plants v33 | 10.1093/nar/gkv1209 |
| Dicots | Populus trichocarpa | 97.02 | ensemble plants v33 | 10.1093/nar/gkv1209 |
| Dicots | Prunus persica | 96.43 | ensemble plants v33 | 10.1093/nar/gkv1209 |
| Dicots | Ricinus communis | 99.93 | TIGR/JCVI v0.1 | 10.1038/nbt.1674 |
| Dicots | Solanum lycopersicum | 97.15 | ensemble plants v33 | 10.1093/nar/gkv1209 |
| Dicots | Solanum tuberosum | 79.75 | ensemble plants v33 | 10.1093/nar/gkv1209 |
| Monocots | Aegilops tauschii | 87.76 | ensemble plants v33 | 10.1093/nar/gkv1208 |
| Monocots | Brachypodium distachyon | 96.89 | ensemble plants v33 | 10.1093/nar/gkv1208 |
| Monocots | Hordeum vulgare | 85.04 | ensemble plants v33 | 10.1093/nar/gkv1208 |
| Monocots | Leersia perrieri | 95.57 | ensemble plants v33 | 10.1093/nar/gkv1208 |
| Monocots | Musa acuminata | 96.29 | ensemble plants v33 | 10.1093/nar/gkv1208 |
| Monocots | Oryza barthii | 94.84 | ensemble plants v33 | 10.1093/nar/gkv1208 |
| Monocots | Oryza brachyantha | 95.43 | ensemble plants v33 | 10.1093/nar/gkv1208 |
| Monocots | Oryza glaberrima | 91.59 | ensemble plants v33 | 10.1093/nar/gkv1208 |
| Monocots | Oryza glumaepatula | 94.64 | ensemble plants v33 | 10.1093/nar/gkv1208 |
| Monocots | Oryza indica | 96.29 | ensemble plants v33 | 10.1093/nar/gkv1208 |
| Monocots | Oryza longistaminata | 91.79 | ensemble plants v33 | 10.1093/nar/gkv1208 |
| Monocots | Oryza meridionalis | 86.43 | ensemble plants v33 | 10.1093/nar/gkv1208 |
| Monocots | Oryza nivara | 95.30 | ensemble plants v33 | 10.1093/nar/gkv1208 |
| Monocots | Oryza punctata | 95.50 | ensemble plants v33 | 10.1093/nar/gkv1208 |
| Monocots | Oryza rufipogon | 94.90 | ensemble plants v33 | 10.1093/nar/gkv1208 |
| Monocots | Oryza sativa | 89.48 | ensemble plants v33 | 10.1093/nar/gkv1208 |
| Monocots | Setaria italica | 97.02 | ensemble plants v33 | 10.1093/nar/gkv1208 |
| Monocots | Sorghum bicolor | 94.31 | ensemble plants v33 | 10.1093/nar/gkv1208 |
| Monocots | Triticum aestivum | 97.88 | ensemble plants v33 | 10.1093/nar/gkv1208 |

| | | | | |
|---|---|---|---|---|
| Monocots | Triticum urartu | 87.49 | ensemble plants v33 | 10.1093/nar/gkv1208 |
| Outgroups | Arabidopsis thaliana | 100 | ensemble v86 | 10.1093/database/baw093 |
| Outgroups | Caenorhabditis elegans | 99,93 | ensemble v86 | 10.1093/database/baw093 |
| Outgroups | Drosophila melanogaster | 99,93 | ensemble v86 | 10.1093/database/baw093 |
| Outgroups | Homo sapiens | 100 | ensemble v86 | 10.1093/database/baw093 |
| Outgroups | Saccharomyces cerevisiae | 100 | ensemble v86 | 10.1093/database/baw093 |
| Outgroups | Strigamia maritima | 92,06 | ensemble metazoa v34 | 10.1093/database/baw093 |
